# Advancing patient-centric care: integrating patient reported outcomes for tolerability assessment in early phase clinical trials – insights from an expert virtual roundtable

**DOI:** 10.1016/j.eclinm.2024.102838

**Published:** 2024-09-24

**Authors:** Christina Yap, Olalekan Lee Aiyegbusi, Emily Alger, Ethan Basch, Jill Bell, Vishal Bhatnagar, David Cella, Philip Collis, Amylou C. Dueck, Alexandra Gilbert, Ari Gnanasakthy, Alastair Greystoke, Aaron R. Hansen, Paul Kamudoni, Olga Kholmanskikh, Bellinda L. King-Kallimanis, Harlan Krumholz, Anna Minchom, Daniel O'Connor, Joan Petrie, Claire Piccinin, Khadija Rerhou Rantell, Saaeha Rauz, Ameeta Retzer, Steven Rizk, Lynne Wagner, Maxime Sasseville, Lesley K. Seymour, Harald A. Weber, Roger Wilson, Melanie Calvert, John Devin Peipert

**Affiliations:** a Clinical Trials and Statistics Unit, The Institute of Cancer Research, London, UK; b Centre for Patient-Reported Outcomes Research (CPROR), Institute of Applied Health Research, University of Birmingham, Birmingham, UK; c Lineberger Comprehensive Cancer Center, Chapel Hill, NC, 27514, USA; d AstraZeneca, Oncology Research and Development, Gaithersburg, MD, USA; e Oncology Center of Excellence, US Food and Drug Administration, Silver Spring, MD, USA; f Department of Medical Social Sciences, Northwestern University Feinberg School of Medicine, Chicago, IL, USA; g Department of Quantitative Health Sciences, Mayo Clinic, Scottsdale, AZ, USA; h Leeds Institute for Medical Research, University of Leeds, St James's University Hospital, Leeds, UK; i RTI Health Solutions, Patient Centered Outcomes Research, Research Triangle Park, NC, USA; j NU Cancer, Newcastle University, Newcastle upon Tyne, UK; k Princess Alexandra Hospital, Cancer Services, Brisbane, Queensland, Australia; l Merck KGaA, Darmstadt, Germany; m Federal Agency for Medicines and Health Products (FAMHP), Brussels, Belgium; n LUNGevity Foundation, Bethesda, MD, USA; o Center for Outcomes Research and Evaluation, Yale New Haven Hospital, New Haven, CT, USA; p Drug Development Unit, Royal Marsden/Institute of Cancer Research, Sutton, UK; q The Association of the British Pharmaceutical Industry, UK; r Canadian Cancer Trials Group (CCTG), Kingston, Ontario, Canada; s Quality of Life Department, European Organisation for Research and Treatment of Cancer, Brussels, Belgium; t Medicines and Healthcare Products Regulatory Agency (MHRA), UK; u Academic Unit of Ophthalmology, Institute of Inflammation and Ageing, University of Birmingham, UK; v Centre for Patient Reported Outcomes Research (CPROR), Institute of Applied Health Research, University of Birmingham, Birmingham, UK; w Veloxis Pharmaceuticals, Cary, NC, USA; x University of North Carolina Gillings School of Global Public Health, Chapel Hill, NC, USA; y Health Canada, Ottawa, Ontario, Canada; z Pfizer Oncology, Global Medical Affairs/Early-Stage Development, Zug, Switzerland; aa Cancer Research Advocates Forum UK, Sarcoma Patient Advocacy Global Network, UK; ab National Institute for Health and Care Research (NIHR) Birmingham Biomedical Research Centre (BRC), University Hospital Birmingham and University of Birmingham, Birmingham, UK; ac NIHR Applied Research Collaboration West Midlands, University of Birmingham, Birmingham, UK; ad NIHR Blood and Transplant Research Unit in Precision Transplant and Cellular Therapeutics, University of Birmingham, Birmingham, UK; ae Birmingham Health Partners Centre for Regulatory Science and Innovation, University of Birmingham, Birmingham, UK; af Division of Oncology, Department of Medicine, University of North Carolina, Chapel Hill, NC, 27514, USA; ag University of Queensland, Faculty of Medicine, Australia; ah Birmingham and Midland Eye Centre, Sandwell and West Birmingham Hospitals NHS Trust, Birmingham, UK; ai National Institute of Health and Care Research (NIHR) Birmingham Biomedical Research Centre, University of Birmingham and University Hospitals Birmingham NHS Foundation Trust, Birmingham, UK

**Keywords:** Patient-reported outcomes, Dose-finding, Phase I, Phase II, Early phase trials, Tolerability, Patient-centred clinical development, Quality of life, Trial designs

## Abstract

Early phase clinical trials provide an initial evaluation of therapies’ risks and benefits to patients, including safety and tolerability, which typically relies on reporting outcomes by investigator and laboratory assessments. Use of patient-reported outcomes (PROs) to inform risks (tolerability) and benefits (improvement in disease symptoms) is more common in later than early phase trials. We convened a two-day expert roundtable covering: (1) the necessity and feasibility of a universal PRO core conceptual model for early phase trials; (2) the practical integration of PROs in early phase trials to inform tolerability assessment, guide dose decisions, or as real-time safety alerts to enhance investigator-reported adverse events. Participants (n = 22) included: patient advocates, regulators, clinicians, statisticians, pharmaceutical representatives, and PRO methodologists working across diverse clinical areas. In this manuscript, we report major recommendations resulting from the roundtable discussions corresponding to each theme. Additionally, we highlight priority areas necessitating further investigation.

## Introduction

Early phase (I/II) clinical trials advance clinical development by providing crucial insights into dose selection for the investigational therapy and into adverse events (AEs) arising from different dosages, the interaction with the human body, and to capture early signals, indicating benefits for patients.^1^, ^2^, ^3^, ^4^ Assessment of a therapy's safety and tolerability has predominantly relied on clinical investigator reporting and interpretation using the Common Terminology Criteria for Adverse Events (CTCAE) or other AE grading approaches that do not include direct reports from patients. A review of ClinicalTrials.gov from 2007 to 2020 showed that PROs were included in only 5.3% (548/10,372) of trials, though their use increased over time, primarily (89.6%) as secondary outcomes.^5^ Compared to patients, clinicians significantly underreport symptomatic AEs.^6^, ^7^, ^8^, ^9^ AEs that are challenging to observe (e.g., fatigue) may be inadequately characterised without direct input from patients, and the cumulative impact of multiple AEs on the patient is not adequately captured by individual AE reporting.^10^ These deficiencies may lead to inaccurate tolerability assessment, incorrect or sub-optimal dose-selection, sub-optimal risk-benefit evaluation, and, ultimately, delays or failures in the clinical development pathway. Moreover, because PROs can be captured in real-time between study visits, omitting them from early phase trials is a missed opportunities to efficiently capture AEs as they arise and intervene with patients to manage AEs, develop supportive and risk minimisation measures, or collect critical information to plan for subsequent trials.^11^

Historically, dose-finding trials in oncology have focused on dose-limiting toxicity (DLT) identified from cycle 1 data, especially in trials utilising rule-based designs like 3 + 3.^12^ The 3 + 3 design involves administering a dose to three patients at a time, escalating the dose if there are no or minimal toxicities, and determining the Maximum Tolerated Dose (MTD) based on observed DLTs within a defined time window. The identified MTD is usually the recommended dose in subsequent testing. These designs have infrequently capitalized on the potential for PROs to enhance dose decisions. A recent review (2016–2022) of published dose-finding oncology trials with PRO analysis found that PROs influenced dose decisions in only 11.4% (4/35) of trials. Three trials utilised PROs only at the end to confirm the tolerability of the recommended phase 2 dose. Notably, only one 3 + 3 trial formally incorporated PROs to inform interim dose decisions, defining a specific PRO score increase as a DLT.^13^ Since 2020, the novel dose-finding model-based and model-assisted designs PRO-CRM, U-PRO-CRM, and PRO-ISO design have emerged, formally incorporating PROs.^14^, ^15^, ^16^ These designs integrate patient- and investigator-reported information to guide interim dose assignment decisions and provide the final dose recommendations. They can also be extended to capture cumulative or late-onset toxicities, as demonstrated in the TITE-PRO-CRM design.^17^ These advancements optimise dose-finding by incorporating patient voice on treatment tolerability, which is crucial for targeted therapies and immunotherapies intended for long-term administration.

Despite limited use in dose-finding oncology trials, a global survey found widespread endorsement from over 100 stakeholders regarding the use of PROs for assessing tolerability and informing dose selection.^18^ However, a significant barrier identified for PRO implementation was the absence of guidance on which PROs should be utilised and implemented. Recent initiatives such as the FDA Optimus Project,^19^ aiming to reform dose optimization in oncology,^20^^,^^21^ and the Methodology for the Development of Innovative Cancer Therapies guidelines,^22^ highlight the growing recognition of the value of incorporating PROs in early phase trials. While the US FDA has advanced a core set of PROs for later phase cancer trials, no such recommendations exist for early phase trials,^23^ though EMA guidance recommends considering PROs early in the development programme, particularly if there is a need to develop a dedicated instrument.^24^

This article discusses integrating PROs in early phase exploratory trials, encompassing phase I dose-escalation studies and first-in-human investigations, as well as dose expansion, dose optimisation, seamless phase I/II and phase II trials. Drawing insights from an expert roundtable, the paper explores two main themes: the feasibility of establishing a universal PRO core conceptual model for assessing tolerability in early phase trials across various disease settings and the utility of PROs in guiding dose decisions and acting as real-time safety alerts.

## Methods

On November 30th and December 1st, 2023, we organised two half-day expert virtual roundtable meetings. We invited representatives from key stakeholder groups to ensure diverse range of perspectives and relevant expertise, including individuals recognised for their publications or expertise in PROs and/or early phase trials. This included patient advocates, regulators, experienced clinical trialists, pharmaceutical industry representatives, and PRO methodologists. One participant coded as a PRO methodologist is a biostatistician who works on oncology trials. Participants with both oncology and non-oncology experience were represented. Pre-meeting materials, detailing objectives and key questions, were sent to participants beforehand. Before exploring each theme, the joint hosts (CY, JDP, LA and MC) presented the following topics: 1) General concepts of phase I and II trials, covering research questions, participants, and trial designs; 2) Defining treatment tolerability from the patient's perspective,^25^ including introduction of the US FDA Core Set of PROs for use in Cancer Trials.^23^ The presenters acknowledged that much of the existing evidence originated from oncology studies but emphasised our intention to assess applicability across disease settings.

The presentation also included key questions to facilitate discussion for each theme, summarized in Box 1.

Box 1 **Key questions for each theme**

**Theme 1**

The need and feasibility of establishing a universal set of PRO core concepts for tolerability assessment:

• Is it feasible and is there a need to develop a PRO Core Outcome Set (COS) to assess tolerability in phase I and II trials?
• If yes, would there be major differences between the COS needs for:
  ◦ Phase I and phase II? How?
  ◦ Oncology and non-oncology trials? How?

**Theme 2**

Practical considerations for incorporating PROs in trial design for tolerability assessment:

• How and for what purpose should PROs be used in early phase settings?
Key prompts
  ◦ How should PROs be used to guide trial design, whether for dose escalation, dose optimisation, or general tolerability assessment to inform PRO strategy in later phase trials?
    ▪ Interim vs end-of trial analysis (and if they should be analysed formally or descriptively)
    ▪ Real time reporting and response (utilising PROs to inform adverse event grading or independent PRO assessment)
    ▪ Standardised vs ad hoc reporting
• What should be the role of PROs in regulatory decision-making in early phase trials?

The hosts led the discussions, starting with key questions and using prompts to guide if needed. Spontaneous, unguided responses were encouraged. Participants who did not offer spontaneous input were called on to increase participation representation. Survey questions were administered via Zoom to quantify agreement with key points during the discussions. Non-presenting hosts took notes, which were compared to aid in summarizing the discussion. The focus was on areas of consensus and where no consensus could be reached. The protocol for these activities was submitted to the Northwestern University (USA) ethics board and deemed not to be human subjects research (#STU00220530).

## Results

Of 32 invited experts to the two-day virtual meeting, 22 participated in the discussions and provided feedback on the draft manuscript (Table 1). Five who were unable to attend also provided input and feedback, while five did not participate at all. The five non-attendee participants included one patient, two academic clinical trialists, one PRO researcher/oncology clinician, and one pharmaceutical company representative. Since they did not participate in the roundtable sessions, they did not vote. They provided critical reviews of roundtable decisions during the manuscript review. We note that the participant count varied between the two days of roundtable discussions, and the number of responses differed across various questions because not all participants voted on every question. This variation arose as some participants joined later or had to leave earlier, while others encountered occasional connectivity issues. Below, we summarise major points emerging from the roundtable discussions, stratified by theme.

**Table 1:** Expert roundtable participants (N = 22).

Patient advocate	2 (9%)
Regulator	4 (18%)
Clinical trialist	5 (23%)
Pharmaceutical representative	4 (18%)
Patient-reported outcomes methodologist	7 (32%)

### Theme 1: model of PRO concepts to assess tolerability in early phase trials

#### Overall benefits and challenges

Participants expressed strong support for using PROs to assess tolerability of new therapeutic agents in early phase trials and believed that PRO data could enhance risk-benefit assessments. Potentially beneficial uses of PROs in phase I trials included aiding clinicians in AE reporting and gathering early information on treatment-emergent AEs to inform future dosing strategies. The latter benefit was deemed particularly crucial to minimise dose modification, interruption, or early treatment discontinuation due to tolerability and safety concerns in later phase trials. Moreover, since PROs can be collected outside clinic visits as AEs occur, they may better characterise AEs than retrospective reports made in study visits in clinic. These advantages also apply to phase II trials. Additionally, patient-reported tolerability in phase I trials can contribute to dose optimisation and scheduling for future testing.^14^^,^^19^^,^^22^^,^^26^ Participants also suggested that PROs in phase II trials would provide critical formative data to support PRO strategies in later phase trials, including measure selection, assessment schedule, and sample size determination. More broadly, one patient advocate remarked that participation in early phase trials may be an emotional experience for patients, and that PROs may play an important role in capturing such experience and its impact on treatment tolerability and acceptability.

**Fig. 1:**
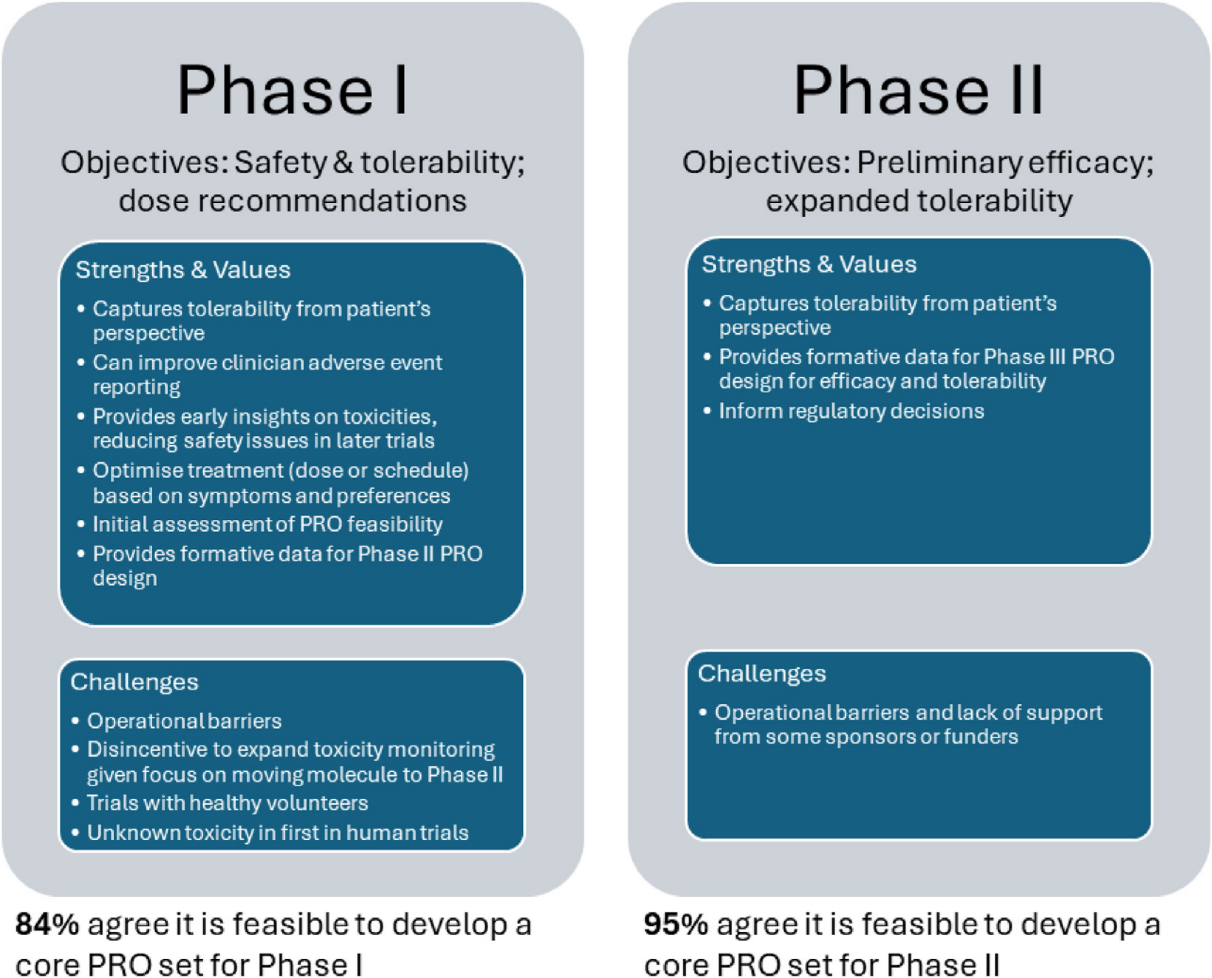
**Benefits and challenges**.

Participants also highlighted challenges in implementing PROs in early phase trials. They noted that communication between departments responsible for late phase studies (which typically have more extensive PRO expertise) and early phase studies may be hindered by existing structural barriers. In addition, phase I trial programs’ strong focus on advancing the experimental treatment to phase II and the high failure rate of new drugs may serve as disincentives to include PROs in phase I studies.^27^ There was also acknowledgement that some phase I studies may include healthy volunteers instead of patients, entailing a somewhat different set of PRO concepts. Finally, in first-in-human trials, the relevant AEs may not be known. One participant noted that cancer trials funded by the National Cancer Institute and conducted by cooperative trials groups in the US do not receive reimbursement for implementation of PROs in Phase II trials unless they are randomised.^28^ Fig. 1 summarizes the benefits and challenges participants attributed to PRO integration in early phase trials. In addition, though the potential to add to patient burden with PRO assessments was mentioned, a patient advocate noted that the benefit of PROs in early phase trials would outweigh the burden of completing them. They emphasised the importance of involving patients and obtaining their input at early planning and trial design stages on integrating PROs.^18^^,^^29^

#### Need and feasibility of a single conceptual model

When asked “Is it feasible and is there a need to develop a PRO core outcome set (COS) to assess tolerability in phase I trials?”, 84% (16/19) of participants agreed that it would be feasible to identify and include a minimum set of PROs in phase I trials. Among those answering “no” (n = 3), the rationale was that institutional barriers may reduce feasibility of implementing PROs though they noted that it would be feasible in the future. When asked the same question about phase II trials, 95% (18/19) agreed it would be feasible.

#### Shared vs separate guidance for phase I and phase II

When participants were asked whether there would be major differences in the PRO needs between phase I and phase II, 44% (7/16) said yes. Participants mentioned that the PRO-related objectives for phase I would likely be exclusively focused on tolerability, whereas phase II trials may encompass an additional focus on collection of data to inform both risks and benefits, which may increase the relevance of PROs capturing disease-related symptoms in phase II trials. In contrast, phase I trials involving healthy volunteers may require a somewhat different set of concepts, where disease symptoms would not be relevant.

#### Shared vs separate guidance for oncology and non-oncology settings

Participants discussed whether a single PRO core outcome set would be applicable in oncology and non-oncology settings. Twelve discussion participants (60%) primarily worked in oncology setting. An oncology pharmaceutical representative noted that some phase II oncology trials could provide pivotal results for regulatory approval, highlighting the potential for PROs in phase I trials to offer formative data for phase II design. Additionally, PROs could support cost-effectiveness analyses relevant for reimbursement. There was also acknowledgement that regulators within and outside oncology seek PRO data to support risk-benefit assessment,^24^ particularly in the US where the 21st Century Cures Act, passed in 2016, requires reports of patient experience with drug submissions.^30^ Pharmaceutical representatives working in non-oncology investigational therapy development programs noted that tolerability as a concept is less commonly used outside of oncology, though there is growing interest. When asked whether a common guidance should be developed for oncology and non-oncology trials, only 31% (5/16) responded yes. However, despite acknowledging different trial cultures, participants felt that the central PRO concepts in oncology would also likely be relevant in non-oncology settings. They acknowledged that specific trials may require more tailored approaches, suggesting a common core with “add-on” sets for specific clinical settings.

##### Recommendation 1

Considering these benefits, challenges, and perceived feasibility, we recommend promoting PRO collection in phase I and phase II trials. Key actions in these settings include fostering collaboration between early and late-stage therapeutic development programs, educating and raising awareness about the importance of PRO integration in early phase trials, involving patients and advocacy groups in early phase trial designs integrating PROs, and encouraging funders to allocate targeted funding for early phase trials to prioritize PRO collection inclusion. Future work could involve investigating and developing guidance for a PRO COS, either shared or separate, for phase I and II trials, as well as in both oncology and non-oncology settings (Table 2).

**Table 2:** Recommendations and actions toward integrating PROs in early phase trials.

Recommendations	Actions
1. Promote PRO collection in early phase I and phase II trials	Foster collaboration and communication between early and late-stage therapeutic development programs to facilitate the integration of PROs in phase I and II trials, which would in turn inform future utilisation in later stage trials.
	Educate and raise awareness among stakeholders about the significance of PROs in early phase I and II trials, emphasising the value of integrating PRO objectives alongside conventional objectives to enhance research quality and patient-centricity.
	Involve patients and patient advocacy groups in the design and conduct of early phase trials, gathering their input on the selection of PRO measures/items and ensuring that their perspectives are captured in the research process.
	Encourage funders to allocate targeted funding for early phase trials that prioritise PRO integration in early phase study designs
2. Adopt the FDA core PRO concepts as an initial step in selecting PRO measures to be collected.	For Phase I: overall side effect impact, symptomatic adverse events
	For Phase II: overall side effect impact, symptomatic adverse events, physical function, role function, and disease symptoms as a core outcome set across oncology and non-oncology settings.
	Develop PRO core outcome sets for phase I and II trials, and consider appropriateness for oncology and non-oncology settings.
3. Conduct additional research into multiple aspects of integrating PROs into early phase trials:	
a. Feasibility and validity of implementing of PROs in early phase trials	Conduct pilot studies that determine feasibility across differing trial settings, implementation challenges potentially encountered by clinical teams when PROs are implemented in real time and ad hoc, with or without real-time alerts.
b. Using PROs to inform interim trial decisions, end-of-trial analysis, and CTCAE grading, considering descriptive and comparative PRO analyses	Conduct additional consensus-building work with diverse stakeholders, including clinical trialists and methodologists, patients, PRO researchers, and regulators

#### Suitability of the FDA core PROs in oncology for early phase trials

**Fig. 2:**
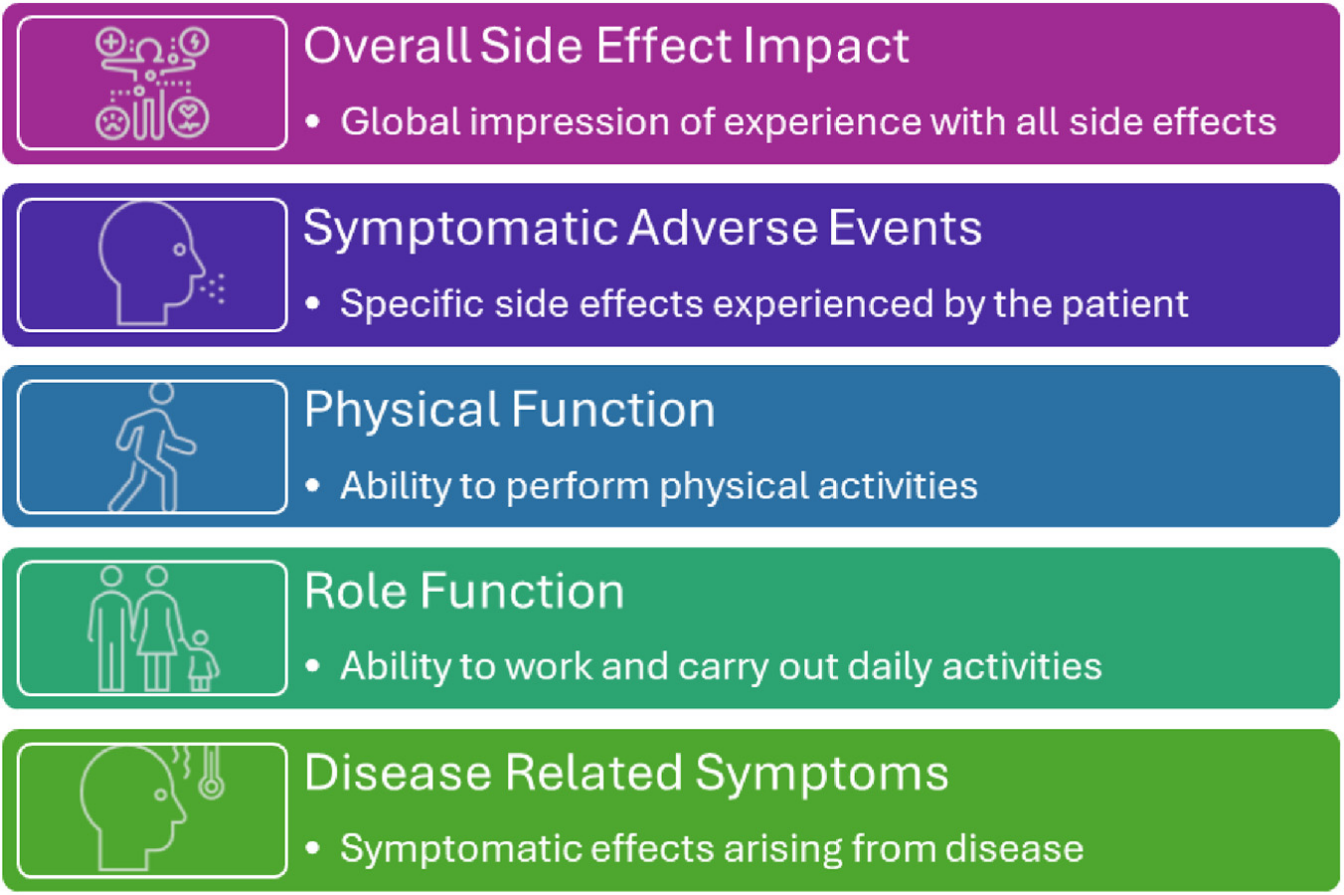
**Core set of PRO conceptual model adapted from US FDA guidance**.

Given the general agreement that a conceptual model to guide use of PROs in early phase trials was feasible, we considered whether the FDA Core PRO concepts would fit this need. In 2021, the US FDA published their draft guidance document, *Core Patient-Reported Outcomes in Cancer Clinical Trials Guidance for Industry*.^23^ This guidance identified core PRO concepts for assessment in cancer trials, including disease related symptoms, symptomatic AEs, overall side effect impact, physical function, and role function (Fig. 2). An earlier version of these core concepts without overall side effect impact and role function was registered with the COMET Initiative^31^ and published.^32^ Among the core PRO concepts, symptomatic AEs, overall side effect impact, and physical function can be considered directly relevant to tolerability assessment, while role function may be considered indirectly relevant. The FDA PRO Core concepts were not designed for early phase trials, but the included concepts were deemed to be very likely relevant to capturing tolerability in early phase trials.^33^

**Fig. 3:**
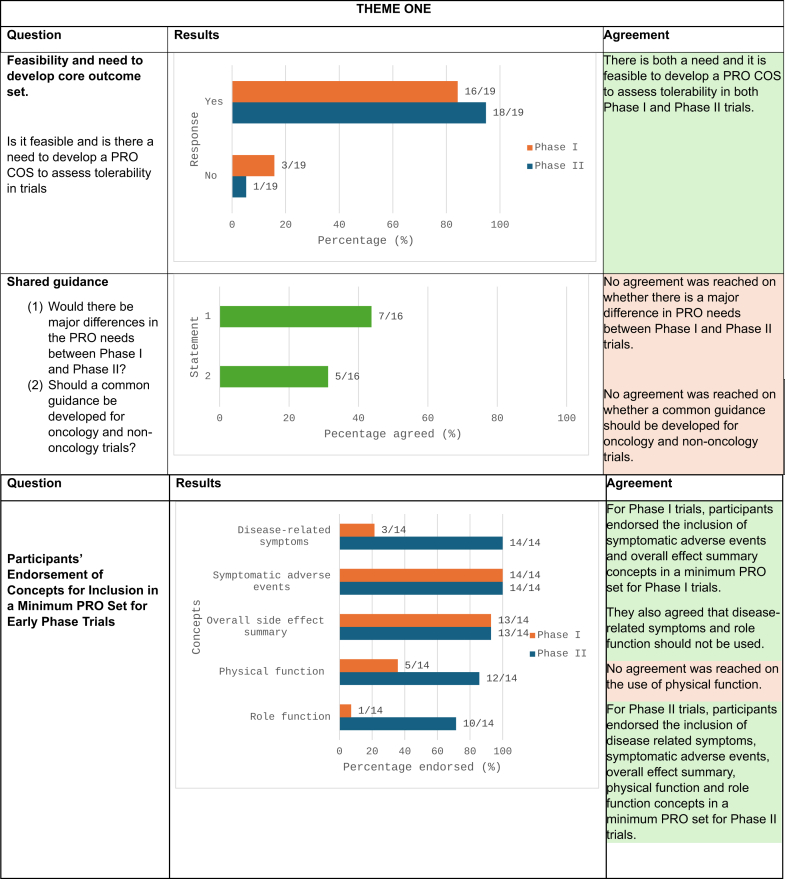

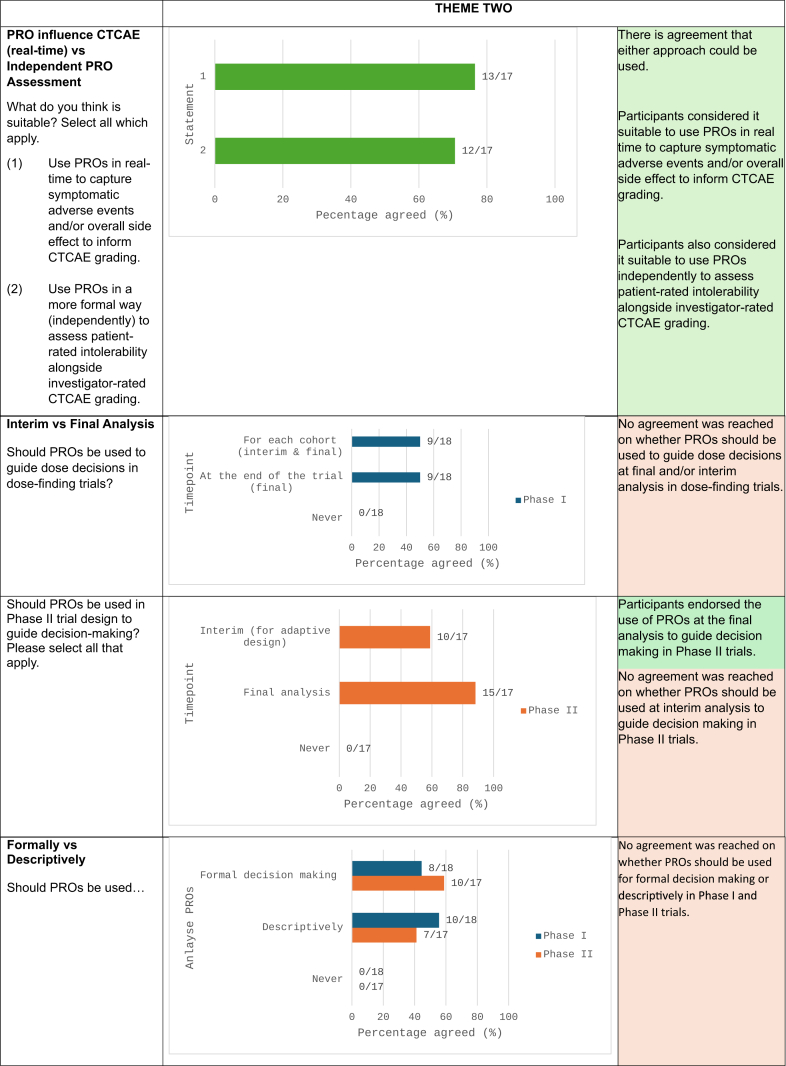
**Poll questions for participants for themes one and two. For illustrative purposes, high level of agreement (≥70% of participants) indicative that consensus is reached are coloured in green, and the rest are coloured in orange (<70% of participants)**.

Participants reviewed the FDA Core PROs in oncology and supported their use in early phase trials, considering them suitable for both phase I and II trials as a starting point. When asked, “What are the minimum set of core concepts which should be included within phase I/II trials (please tick all which apply)” (asked separately about phase I and II) and given a choice of the concepts from the FDA Core PROs as options, there was high agreement that symptomatic AEs and overall side effects impact summary should be included in both phase I and II (Fig. 3). Fewer participants prioritized physical function, role function, and disease-related symptoms for phase I trials, but the endorsement rate for all FDA Core PROs was high for phase II trials. Participants acknowledged key benefits of starting with the FDA Core PROs for Cancer Trials include its flexibility and ease of tailoring to different study needs, the capture of key concepts with only 1–2 items (questions), and its existing use in FDA-reviewed phase III trials. They also identified areas requiring additional research for implementing FDA Core PRO concepts in early phase trials. These include more granular examination of the importance of tolerability in different settings depending on the investigational therapy and disease. Despite this, there was agreement that all concepts would be relevant in most therapeutic areas. Finally, discussions emphasized the need for further specification of endpoints depending on whether descriptive or decision-informing analyses are planned.

##### Recommendation 2

Adopt the FDA core PRO concepts - overall side effect impact, symptomatic AEs, physical function, role function, and disease symptoms–across oncology and non-oncology settings for phase II trials. For phase I trials, prioritize a smaller core set using the FDA core PRO concepts as a basis, that include the concepts of overall side effect impact and symptomatic AEs.

### Theme 2: practical recommendations for integrating PROs in early phase trials

#### PROs influence CTCAE (real-time) vs independent PRO assessment

The panel deliberated on two approaches for phase I and II jointly: (1) using PROs capturing AEs and/or overall side effect impacts to inform investigator-reported AE grading, potentially by promptly signalling red flags (such as severe PRO scores) to investigators in real-time, or (2) assessing both investigator and patient reports independently, such that PROs complement but do not influence investigator-reported AE gradings. The panel had the option to select either or both approaches and voted 13/17 for (1) and 12/17 for (2) (Fig. 3).

Both approaches were considered viable, contingent upon specific circumstances. The feasibility of the first approach would depend on operational and logistical considerations specific to the organization, as well as the study team's willingness and resources to manage real-time flagging of PROs for safety monitoring. The second approach may be preferable in cases where it is desirable to maintain independence between the two outcomes without any potential influence. For instance, in sensitive topics where there could be a strong social desirability bias influencing patient reporting if results were shared with clinicians. Additionally, patients' reporting behaviour may be altered as they are often concerned that reporting severe symptoms may result to changes to their dosage, dose interruptions or treatment withdrawal, potentially altering their reporting behaviour.^34^

#### Interim vs final analysis

The panel discussed whether PROs should guide interim decisions, or be reserved solely for final analysis. Participants strongly agreed PROs should play a role in decision-making in general, though refinement, clarity, and consensus of when to do so is required. For dose-finding trials, no consensus was reached on whether PROs should guide dose-decisions at both the interim and final assessments or only at the final analysis, with a vote distribution of 50%–50%. For phase II trials, the majority (88.2%) felt that PROs should be used at the final analysis, while for trials utilising adaptive designs, 58.8% supported using PROs to guide interim decisions (Fig. 3). Participants noted that utilising PROs to guide interim decision making would be beneficial. However, they expressed concerns about the logistical challenges, particularly in situations where decisions need to be made rapidly, such as patient assignments to the next dose level in dose-finding trials.

#### Formally vs descriptively

The panel further deliberated if PROs should be incorporated formally within the trial design, or be used descriptively only to inform decision making. No consensus was reached on whether the incorporation of PROs for this purpose, should be formal vs descriptive use, with a vote distribution of 44%–56% for dose-finding trials and 59%–41% for phase II trials (Fig. 3).

#### Standardised vs ad hoc reporting

In this context, standardised reporting refers to protocol-defined time-points for PRO assessments, whereas ad hoc reporting refers to additional PRO assessments triggered by trial participants experiencing a change in outcomes. The concept of ad hoc reporting was recognised as still exploratory with potential advantages particularly in early phase settings, although its feasibility remained uncertain. Previous research by Basch and colleagues demonstrated that real-time alerts and monitoring led to reduced hospitalizations and improved survival.^35^^,^^36^ Though somewhat different than ad hoc reporting, these results may indicate promise for the ability of ad hoc PRO reports to have clinical value, though this would require testing. No formal vote was conducted on this matter.

#### Regulators’ perspectives

Our regulatory participants emphasised the importance of adequately collecting PROs data and gaining a clearer understanding of their potential value in decision-making. They recommended starting with a more conservative, primarily evidence-generating approach before proceeding to more formal integration, such as interim decisions. They strongly support incorporating the patient voice early on, particularly regarding patient-reported tolerability, which can impact dosing, and compliance, which would add value to clinical development they also highlighted the need to build the evidence base for use in early phase settings to enable regulatory acceptance. Nonetheless, they recognised the value of using information in real-time or on an interim basis.

##### Recommendation 3

Conduct additional research into multiple aspects of integrating PROs into early phase trials, including their feasibility, validity, and their use in informing decisions across different study designs. Key actions to address these recommendations include conducting pilot studies to determine feasibility and validity of PRO data collection in phase I and II trials across different trial settings and approaches to PRO implementation, similar to PRO feasibility assessments performed in the real world setting.^37^ For example, piloting differing assessment schedules, implementing real time alerts, and ad hoc reporting are needed before specific study design recommendations can be made. To refine how PROs can support decisions about investigational therapy tolerability, and/or dose-finding/optimization, additional consensus building activities with diverse stakeholders are needed. For example, questions regarding whether PRO data should support AE grading in real-time, or only utilized at the trial's conclusion, and whether PRO data should be summarised descriptively or formally guide decision-making, requires further attention (Table 2). Additionally, work to understand how to weight the relative importance of reports from patients and clinicians in tolerability assessment is needed.

## Discussion

PROs are valuable for assessing risks and benefits of emerging treatments in clinical trials,^38^ but guidance is limited for early phase trials. To address this, an international group of experts representing multiple stakeholder types participated in a two-day roundtable and identified ways that PROs can generate substantial value in both phase I and II trials. Based on these discussions, we generated recommendations for implementing PROs in early phase trials and highlighted key areas for future research where consensus was lacking.

Advancing PRO use in early phase trials will require more pilot and demonstration projects. To our knowledge, few prospectively designed studies have explored how PROs might affect AE assessment in early phase settings.^39^ Veitch and colleagues collected patient-rated AEs using the PRO-CTCAE along with standard physician rated AEs in a phase I trial of solid tumor patients, though the PRO assessments were not used to inform the physician-rated AEs.^9^ This study found considerable disagreement between PRO and non-PRO AE ratings, indicating the strong potential for PRO-informed AE ratings to differ from a purely physician-rated approach. Ideally, a prospective study comparing PRO-informed and non-PRO-informed physician-rated AEs could be conducted to determine the feasibility and impact of this approach. A current trial is examining whether PROs can improve the reliability of AE grading, and the results are expected to be informative for PRO implementation in early phase trials.^40^ In addition, greater specification of how PROs would be used to inform AE ratings and decision-making is required. For example, the PRO and physician ratings may be made separately and then evaluated through a formal or informal review process. Finally, the potential advantages and challenges of implementing PROs in more complex designs testing multiple hypotheses among diverse subgroups with small sample sizes (e.g., basket and umbrella trials) requires further exploration.

One of the roundtable's goals was to determine whether separate guidance would be required for oncology vs non-oncology trials. Though only one discussion topic within Theme 1 specifically addressed oncology vs non-oncology trial settings, we acknowledge that the majority of participants (60%) primarily worked in oncology setting. Some participants noted that the concept of tolerability, especially as it applies to PROs, is most familiar in oncology presently. In addition, a key take-away of our work is agreement on primarily considering core PRO concepts, adapted from the FDA guidance for registration trials for anti-cancer therapies, as the basis for a minimum set of PRO concepts for early phase trials. This approach is particularly relevant to early phase cancer trials, as it provides a consistent framework for examining PROs across different trial phases. By establishing this link between early and late phase trials, researchers can gain valuable insights into the patient experience and the impact of cancer treatment over time. We note that all the recommendations made in this paper are relevant for considering in both oncology and non-oncology trials; however, further evaluation may be necessary before applying the FDA core PRO conceptual framework to non-oncology settings.

Investigators from an academic cancer centre previously developed a phase I trial-tailored PRO assessment, bolstering optimism about the feasibility of generating a PRO core for this setting. Retzer and colleagues laid-out a multi-stage strategy for PRO assessment in early phase trials which specifies identifying aims, objectives, and concepts of interest as the first step, after which key outcomes are identified related to the rationale for assessment, including specific PRO measures.^41^ An early phase trialist wishing to include PROs should identify which of the core PRO concepts recommended here are most appropriate for their study, then select specific PRO measures accordingly. The FDA's Guidance^23^ identified the Functional Assessment of Cancer Therapy (FACIT) GP5 item (“I am bothered by side effects of treatment”) and the European Organisation for Research and Treatment of Cancer (EORTC) Q168 (“To what extent have you been troubled with side-effects from your treatment”) as options for capturing overall side effect impact, and the PRO-CTCAE, and FACIT and EORTC item libraries as options for capturing symptomatic AEs. Several PRO measurement systems and item libraries, including cancer specific FACIT and EORTC, but others including the Symptom Burden Questionnaire^42^ and PROMIS^43^ offer PRO measures of physical function, role function, and disease symptoms. Overall side effect impact PROs are likely relevant across many different toxicity profiles and are typically captured with a single overall question, leading to potential efficiencies in early phase settings where a treatment's full AE profile is unknown. While including questions for all potential toxicities can increase patient burden,^41^^,^^44^^,^^45^ focusing on common toxicities (e.g., nausea, diarrhoea, and fatigue in oncology) strikes a good balance. Addition of a free text option can also provide valuable insights into unanticipated side effects.^46^ A comprehensive PRO strategy with clearly defined PRO objectives (e.g., using the estimand framework)^47^ would include specification of assessment timepoints, administration approaches (e.g., paper vs electronic), and ethical agreements.^11^^,^^34^^,^^48^

Our work has strengths and limitations. Key strengths include integration of multidisciplinary expertise, fostering rich contextual discussions and diverse and nuanced perspectives, and an efficient and flexible roundtable approach, allowing dynamic discussion between participants, immediate feedback in response to key questions, and the ability to pursue emerging topics as they arose. Additionally, we employed structured polls to quantify opinions about key issues from participants on the spot. Moreover, a significant strength was patient involvement, including those with extensive experience and recent lived experiences of different chronic diseases with treatment burden. Limitations included having predominantly participants from the US, Europe and Canada (with only one participant from Australia), potentially limiting global representativeness despite reflecting current leadership in the field. The broad focus on both oncology and non-oncology may not fully capture the diverse needs and priorities of specific clinical areas that are not represented. Moreover, the joint hosts presented an introduction to the discussion topics and existing research, which focused largely on oncology since that field has been the primary area of focus in use of PROs in early phase trials to date. Though this presentation could have influenced participants' responses, it was necessary to inform the discussion. Importantly, the joint hosts deliberately refrained from expressing their own preferences or views on a preferred approach. Other limitations included the absence of more structured processes with more participants like the Delphi method or nominal group technique, which might affect the rigour and reliability of the recommendations, and the potential influence of strong opinions on group's consensus. To minimise this potential source of bias, we anonymized the polls for key recommendations. This roundtable discussion, involving multidisciplinary international experts, is an initial step in addressing the emerging field of PROs in early phase trials. To support comprehensive consensus-based recommendations, we must first elicit input on major concepts, explore key points to address, and expand the evidence base. We recognise the necessity for additional, more representative consensus work, like a Delphi exercise using the EQUATOR approach for guidance generation. We plan to conduct a Delphi exercise in the near future, with some leading authors already working on related initiatives, including the development of a PRO core outcome set^49^ and PRO analysis recommendations for dose-finding oncology trials. Finally, though we had a diverse set of participants, we were unable to compare responses to the polling questions by stakeholder group because the individual responses to each question could not be linked to their respective stakeholder groups.

Since the expert roundtable meeting, an FDA workshop has highlighted the value of assessing tolerability from the patient perspectives in early phase cancer trials.^50^ This suggests that some regulatory agencies have greater recognition of the importance of collecting PROs in cancer trials, as they have outlined ways that this information will be considered in benefit-risk evaluation.

In conclusion, integrating PROs in early phase trials represents a critical next step in patient-focused clinical development and ensures that emerging treatments reflect patients’ experiences and priorities. While PROs are used to assess treatment tolerability in later phase trials, their application in earlier phase trials, where tolerability and getting the dosing strategy right are vital, remains underdeveloped. The expert roundtable recommendations presented here will advance the ability to use PROs in early phase trials directly, or point to areas of additional research. Ultimately, alignment of investigational therapy developers, regulators, and clinical trialists will be needed to realise this vision, and multidisciplinary efforts like ours provide a useful model for achieving that.

## Contributors

CY, JDP, OLA and MC conceived the idea for the expert roundtable discussion, prepared the materials and were responsible for investigation and methodology. CY and JDP wrote the first draft of the manuscript with input from MC and OLA. EA, CY and JDP handled data curation, analysis and visualisation. EA and CY have access to the raw data. All authors contributed to the roundtable discussions or provided feedback. All authors critically reviewed the manuscript and approved the final version.

## Declaration of interests

C.Y. has received consulting fees from Faron Pharmaceuticals and an honoraria from Bayer.

O.L.A. reported receiving grants from the National Institute for Health and Care Research (NIHR) Birmingham Biomedical Research center, NIHR Applied Research Collaboration West Midlands, NIHR Blood Transplant Research Unit (BRTU), UK Research and Innovation (UKRI), Health Foundation, Gilead, Anthony Nolan, GlaxoSmithKline, Merck, and Sarcoma UK. OLA declares personal fees from Gilead Sciences Ltd, Merck, and GlaxoSmithKline, Innovate UK outside the submitted work.

E.B. has received payments as a scientific advisor for Navigating Cancer, AstraZeneca, Resilience, N-Power Medicine, and Verily.

J.B. is an employee of AstraZeneca, with ownership interest in AstraZeneca and is engaged by Evinova, a separate healthtech business within the AstraZeneca group.

A.H. has Research funding (paid to institution): Advancell, BMS, MSD, Macrogenics, Tyra Biosciences, Janssen, Seagen, Aveo, Roche/Genetech; Consulting (paid personally): MSD, Pfizer, Eisai, Astellas, Bayer.

P.K. has shares in Merck Healthcare KgaA and is a Full-time employee of Merck Healthcare KgA.

B.K.K. has grants paid direct to organisation from AstraZeneca, Boehringer Ingelheim, Bristol Myers Squibb, Jazz Pharma, Genentech, Eli Lilly, Janssen, Takeda, Dachii Sankyo, Blueprint, Medicines, Janssen, Amgen, Seagen, Manta Cares and personal consultancy fees from Eli Lilly, BMS, AbbVie, Shionogi.

H.M.K. has received grants unrelated to the work from the American Heart Association to Jansson; Agency for Healthcare Research and Quality to Kenvue; National Institutes of Health to Novartis; Centers for Medicare & Medicaid Services to Pfizer and Centers for Disease Control and Prevention. These grants and contracts are unrelated to the work above and are through Yale University or Yale New Haven Hospital. Received consulting fees from Massachusetts Medical Society (to Co-Editor, Journal Watch Cardiology); UpToDate (as Section Editor) and Ensight (unpaid advisor). Payment or honoraria for lectures, presentations, speakers’ bureaus, manuscript writing or educational events would have received occasional travel expenses and/or honoraria to speak at various educational/academic venues. Received travel expenses and/or honoraria to speak at various educational/academic venues. Stocks in Element Science and Identifeye.

A.M. has served on advisory boards for Janssen Pharmaceuticals, GSK, Merck Pharmaceuticals, Takeda Pharmaceuticals, MSD Pharmaceuticals, Faron Pharmaceuticals, Pfizer Pharmaceuticals, AZ, Genmab Pharmaceuticals and Immutep Pharmaceuticals. Has received honoraria from Chugai Pharmaceuticals, Faron Pharmaceuticals, Merck Pharmaceuticals, GSK, Seagen, Takeda Pharmaceuticals and Janssen Pharmaceuticals. Has travel support from Amgen Pharmaceuticals and Janssen Pharmaceuticals. Has received research funding from Astex Pharmaceutical, Merck Pharmaceuticals and MSD.

J.P. is a Patient representative on CCTG.

S.R. Research grants with Medical Research Council, NIH/NEI, NIHR outside permitted work.

A.R. reported receiving grants from National Institute for Health Research (NIHR) Birmingham Biomedical Research Centre (BRC) paid to University of Birmingham and has funding outside the submitted work from NIHR, Birmingham City Council, ACCELERATE, Office of Health Disparity and Inequality. Support for attending meetings from Outsourcing in Clinical Trials UK/Ireland as invited speaker. Honoraria from Wellcome Sanger Institute Representative Research Strategy Advisory Group and ACCELERATE (Innovations for Children and Adolescents with Cancer) International Patient-Reported Outcomes Working Group—Core Group Member and Work-package Lead (unpaid).

S.R. is employed by Veloxis Pharmaceuticals, Inc. Stock in the following companies: Bristol Myers-Squibb, Gilead Sciences, IGM Biosciences, Merck & Co, Pfizer.

L.I.W. has received consulting fees from Celgene/Bristol Myers Squibb; Connect Multiple Myeloma registry, member Scientific Steering Committee.

L.K.S. reports funding to institution from AZ, Bayer, Roche, GSK, Treadwell, REPARE, Novartis, Janssen, MERCK. Shares: AZ.

H.A.W. is employed by Pfizer AG, Switzerland and owns non-voting shares of Roche Ltd.

M.J.C. is the Director of Birmingham Health Partners Centre for Regulatory Science and Innovation and Centre for Patient Reported Outcomes Research and a National Institute for Health and Care Research (NIHR) senior investigator; has received funding from Anthony Nolan, European Regional Development Fund-Demand Hub and Health Data Research UK, Gilead, GSK, Janssen, Macmillan Cancer Support, Merck, NIHR, NIHR ARC WM, NIHR Birmingham BRC, NIHR BTRU Precision and Cellular Therapeutics, UCB Pharma, UKRI, and UK SPINE; and has received consultancy fees from Aparito, Astellas, Boehringer Ingelheim, CIS Oncology, Daiichi Sankyo, Gilead, Glaukos, GSK, Halfloop, Merck, Patient-Centered Outcomes Research Institute, Pfizer, Takeda, and Vertex Pharmaceuticals Incorporated, outside of the submitted work.

The views and opinions expressed in this publication are those of the individual co-authors and may not be understood or quoted as being made on behalf of or reflecting the position of any organisation, committee, working party or group with which the co-authors are affiliated.
