# Supplementary material for: Advancing patient-centric care: integrating patient reported outcomes for tolerability assessment in early phase clinical trials – insights from an expert virtual roundtable
Source: eClinicalMedicine. 2024 Sep 24;76:102838. doi: 10.1016/j.eclinm.2024.102838 (PMC11462221; doi:10.1016/j.eclinm.2024.102838)
Supplement: Supplementary [file mmc1.docx]

**Appendix**

**Zoom Poll Questions**

**Day 1**

Is it feasible and is there a need to develop a PRO COS to assess tolerability in Phase I trials? (Yes/No)

Is it feasible and is there a need to develop a PRO COS to assess tolerability in Phase II trials? (Yes/No)

Would there be major differences in the PRO needs between Phase I and Phase II? (Yes/No)

Should a common guidance be developed for oncology and non-oncology trials? (Yes/No)

**Day 2**

What are the ESSENTIAL minimum set of core concepts which should be included within Phase I trials (please tick all which apply)

- Disease-related symptoms
- Symptomatic adverse events
- Overall side effect summary
- Physical function
- Role Function

What are the ESSENTIAL minimum set of core concepts which should be included within Phase II trials (please tick all which apply)

- Disease-related symptoms
- Symptomatic adverse events
- Overall side effect summary
- Physical function
- Role Function

What do you think is suitable? Select all which apply

- Use PROs in real-time to capture symptomatic adverse events and/or overall side effect to inform CTCAE grading.
- Use PROs in a more formal way (independently) to assess patient-rated intolerability alongside investigator-rated CTCAE grading.

**Phase I/Dose-finding Trials**

Should PROs be used to guide dose decisions in dose-finding trials?

- For each cohort (interim & final)
- At the end of the trial (final)
- None of the above

Should PROs be used…

- Formally for decision-making (incorporated within the trial design)
- Descriptively
- None of the above

**Phase II**

Should PROs be used in Phase II trial design to guide decision-making? Please select all that apply.

- Interim (for adaptive design)
- Final analysis
- None of the above

Should PROs be used…

- Formally for decision-making (incorporated within the trial design)
- Descriptively
- None of the above
